# Electronic health record–based surveillance of temporal and spatial clustering of carbapenem-resistant organisms in an intensive care unit at a tertiary university hospital

**DOI:** 10.1016/j.infpip.2026.100613

**Published:** 2026-10-20

**Authors:** David Odada, Reena Shah, Farah Ebrahim, Hellen Munyi, Annastacia Mbithi, Jasmit Shah, Revathi Gunturu

**Affiliations:** a Department of Nursing, Aga Khan University Hospital, Nairobi, Kenya; b Department of Quality, Aga Khan University Hospital, Nairobi, Kenya; c Department of Internal Medicine, Aga Khan University Hospital, Nairobi, Kenya; d Department of Pathology, Aga Khan University Hospital, Nairobi, Kenya

**Keywords:** Outbreak detection, Surveillance, Electronic health medical, Carbapenem-resistant organism

## Abstract

**Background:**

Intensive care units (ICUs) are at high risk for healthcare-associated infections caused by Gram-negative carbapenem-resistant organisms (GN-CROs). Timely recognition of unusual resistance patterns and potential transmission clusters can be challenging where resources are limited. This study aimed to use electronic health record (EHR)–based surveillance to detect and characterize temporal and spatial clustering of GN-CROs in an adult ICU.

**Methods:**

We conducted a retrospective observational study using microbiological and clinical data from ICU patients admitted between January 2023 and December 2024. Positive cultures for GN-CROs were included. Potential clusters were initially defined as two or more isolates of the same species with identical resistance profiles identified in different patients within 20 days. We extracted data from the EHR and managed it in Research Electronic Data Capture. Temporal trends were assessed using Shewhart control charts, statistical exceedance was evaluated using Poisson probability thresholds, and spatial clustering was examined based on bed locations. Analyses were performed using R statistical software (Vanderbilt University, Nashville, Tennessee, USA) (version ≥4.0).

**Results:**

Of 353 Gram-negative organisms isolated, 18% (63/353) were carbapenem resistant. *Klebsiella pneumoniae* accounted for 41% (26/63) and *Acinetobacter baumannii* for 27% (17/63) of resistant isolates. Eight temporal clusters exceeded the expected baseline, with several signals crossing predefined statistical process control limits. Recurrent *K. pneumoniae* clustering demonstrated sustained elevations with spatial overlap across ICU bed locations, representing epidemiological signals of potential clustering and transmission.

**Conclusions:**

EHR-based surveillance identified epidemiologically meaningful clusters, triggering transmission investigations. Integrating temporal, spatial, and statistical methods offers a feasible approach for outbreak triggers where molecular typing is unavailable.

## Introduction

Carbapenem-resistant Gram-negative organisms, including carbapenem-resistant Enterobacterales (CRE), carbapenem-resistant *Acinetobacter baumannii* complex, and carbapenem-resistant *Pseudomonas aeruginosa*, are important causes of healthcare-associated infections and pose a major challenge to infection prevention. The increasing use of carbapenems and the emergence of CREs pose a significant global public health threat, with substantial associated morbidity and mortality. Early identification of changing resistance patterns and potential transmission clusters is therefore essential for timely investigation and intervention [1], [2], [3], [4].

Despite advances in molecular surveillance, access to advanced diagnostic and surveillance technologies remains limited in many healthcare settings. These limitations highlight the value of approaches that leverage routinely collected clinical and microbiological data to strengthen surveillance and facilitate early detection of potential transmission clusters [5], [6], [7], [8]. Standardized surveillance approaches are essential for identifying healthcare-associated infections and emerging transmission patterns, but their effectiveness depends on the timely integration of clinical, microbiological, and patient-location data. Fragmented or manual surveillance processes can make it difficult to link microbiological findings with patient movement and spatial patterns, potentially delaying recognition of transmission clusters [9], [10].

Intensive care units (ICUs) are particularly vulnerable to acquisition and transmission of resistant organisms because critically ill patients frequently require invasive devices and procedures, have prolonged hospital stays, and are exposed to broad-spectrum antibiotics [1]. Electronic health records (EHRs) provide an opportunity to integrate clinical, microbiological, and patient-location data to support automated surveillance and timely identification of antimicrobial-resistant organisms and potential transmission clusters. Linking microbiology results with admission and transfer dates, bed location, procedures, and other relevant clinical variables can facilitate continuous surveillance and timely detection of temporal and spatial patterns that may be difficult to identify through conventional manual methods. [11], [12]. Such approaches can support the identification of potential clusters and generate signals for further epidemiological investigation while also providing information that can guide targeted infection prevention and control (IPC) interventions [10], [13]. Despite the increasing availability of EHR systems, evidence on their application to automated surveillance of carbapenem-resistant organisms (CRO) in African healthcare settings remains limited [14]. In particular, the potential of routinely collected EHR data to identify temporal and spatial clustering of resistant organisms within high-risk areas such as adult ICUs remains insufficiently characterized [15]. This study aimed to use automated EHR-based surveillance to detect and characterize the epidemiology, resistance patterns, and temporal and spatial clustering of CROs in an adult ICU at a tertiary hospital in Kenya between January 2023 and December 2024.

## Methods

This retrospective observational study was conducted at the Aga Khan University Hospital, Nairobi, a 300-bed tertiary care teaching hospital with an 11-bed adult ICU and an EHR system. Routinely collected surveillance data from the IPC microbiology repository were retrospectively linked to EHR data using unique patient identifiers. Microbiology culture reports from January 2023 to December 2024 were linked to patient-level clinical and epidemiological information. Microbiology and patient-level EHR data were extracted from the MEDITECH EHR using predefined data fields. Microbiology records were linked to patient encounter records using the unique patient identifier. Admission, discharge, transfer, and ICU location data were extracted retrospectively using encounter timestamps and location codes. The resulting datasets were exported for data cleaning, linkage, and analysis and filtered to include only cultures collected during active ICU admissions, verified using admission–discharge timestamps and location codes. The datasets were then merged and managed within a structured Research Electronic Data Capture database, and a second reviewer independently verified the data to resolve discrepancies and ensure accuracy.

Surveillance focussed on four priority pathogens commonly associated with ICU-acquired infections: *Escherichia coli*, *Klebsiella pneumoniae*, *A. baumannii*, and *P. aeruginosa*. Carbapenem resistance was determined using categorical antimicrobial susceptibility testing results reported by the microbiology laboratory. Isolates categorized as resistant or intermediate to at least one carbapenem agent (imipenem, meropenem, doripenem, or ertapenem) according to the applicable Clinical and Laboratory Standards Institute breakpoints were classified as carbapenem non-susceptible. Case classification was as per the Centers for Disease control and Prevention (CDC) National Healthcare Safety Network (NHSN) Patient Safety Component Manual criteria for multi-drug-resistant organisms (MDROs) and device-associated infections. NHSN laboratory-identified (LabID) rules were used to identify non-duplicate isolates, with the first Gram-negative carbapenem-resistant organism (GN-CRO) isolate per patient, organism, and location within a calendar month classified as a LabID event. Additional isolates during the same month were considered duplicates unless obtained from blood. Blood isolates were evaluated using a 14-day look-back period across calendar months and were excluded as duplicates if an identical organism had been isolated within the prior 14 days; otherwise, they were counted as unique LabID events (Figure 1).

**Figure 1:**
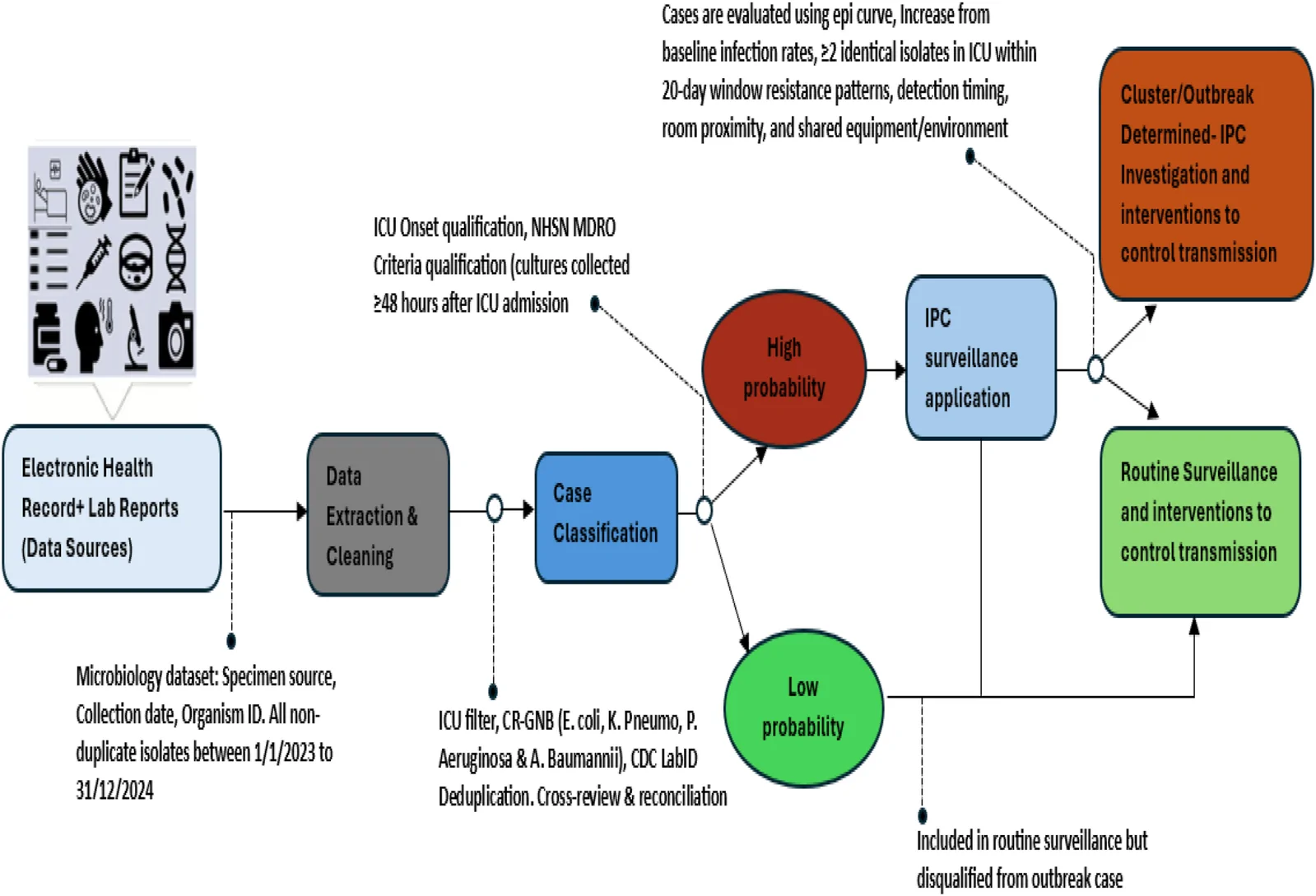
Electronic surveillance algorithm approach to identify MDRO cluster. ICU, intensive care unit; NHSN, National Healthcare Safety Network; MDRO, multi-drug-resistant organism; IPC, infection prevention and control; CDC, Centers for Disease Control and Prevention; CR-GNB, Carbapenem-Resistant Gram-Negative Bacteria.

Potential epidemiological signals of clustering were flagged when two or more isolates of the same species with identical resistance profiles were identified within 20 days in the ICU. Clustering was further evaluated using spatial–temporal permutation analysis, comparisons with historical baseline detection rates, and spatial mapping of ICU bed locations was used to assess the geographic distribution and spatial proximity of clustered isolates. Potential epidemiological relatedness was explored using organism identity, timing of detection, and overlapping ICU stays. Because molecular typing was not performed, these analyses were considered epidemiological signals of potential clustering rather than confirmation of transmission or genetic relatedness.

Descriptive statistics were used to summarize patient characteristics, microbiological findings, and resistance patterns. Shewhart control charts were used for temporal trends, and spatial–temporal permutation analysis was applied to identify statistically significant space–time clusters representing potential epidemiological signals of clustering. Organism-specific isolate counts were evaluated using a Poisson model with a prespecified reference rate of λ = 1 isolate per month as an operational analytical reference. Under this model, the probability of observing exactly three and four isolates in a month was approximately 0.061 and 0.015, respectively. These probabilities were used as an analytical indicator of increasingly unusual monthly counts and to identify signals warranting further epidemiological assessment, rather than as evidence of confirmed transmission. Multi-variate logistic regression models were constructed to identify independent predictors of GN-CRO. Analyses were performed using R statistical software (version ≥4.0).

## Results

Of the 353 Gram-negative organisms isolated from ICU clinical cultures over the two-year study period, 63 (18%) were carbapenem resistant. *K. pneumoniae* accounted for the largest proportion (41%), followed by *A. baumannii* (27%), *E. coli* (21%), and *P. aeruginosa* (11%) (Figure 2). Across all specimen types, *K. pneumoniae* had the highest resistance proportion (45%). (Figure 3).

**Figure 2:**
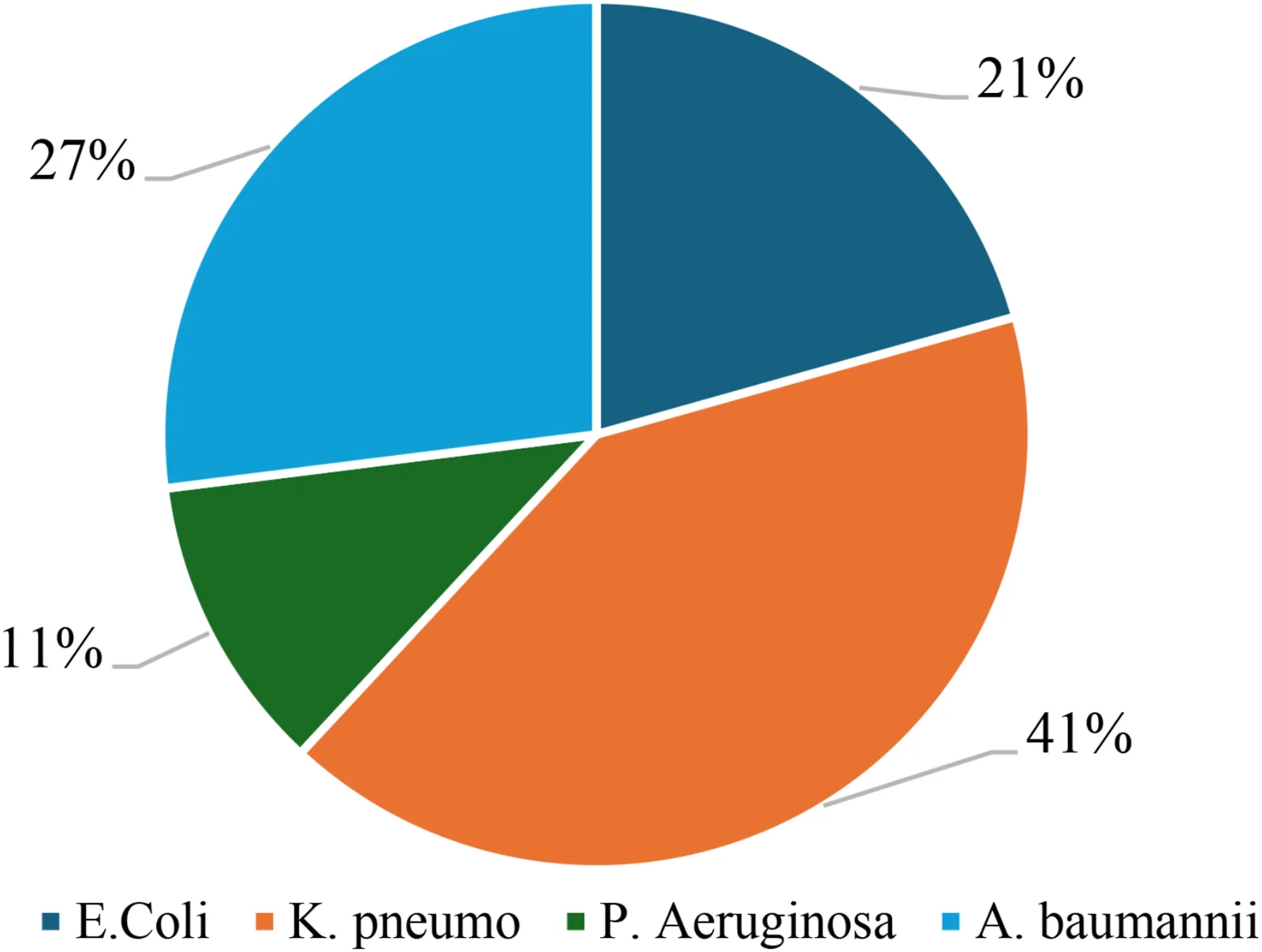
Resistant Pathogens in the ICU. ICU, intensive care unit.

**Figure 3:**
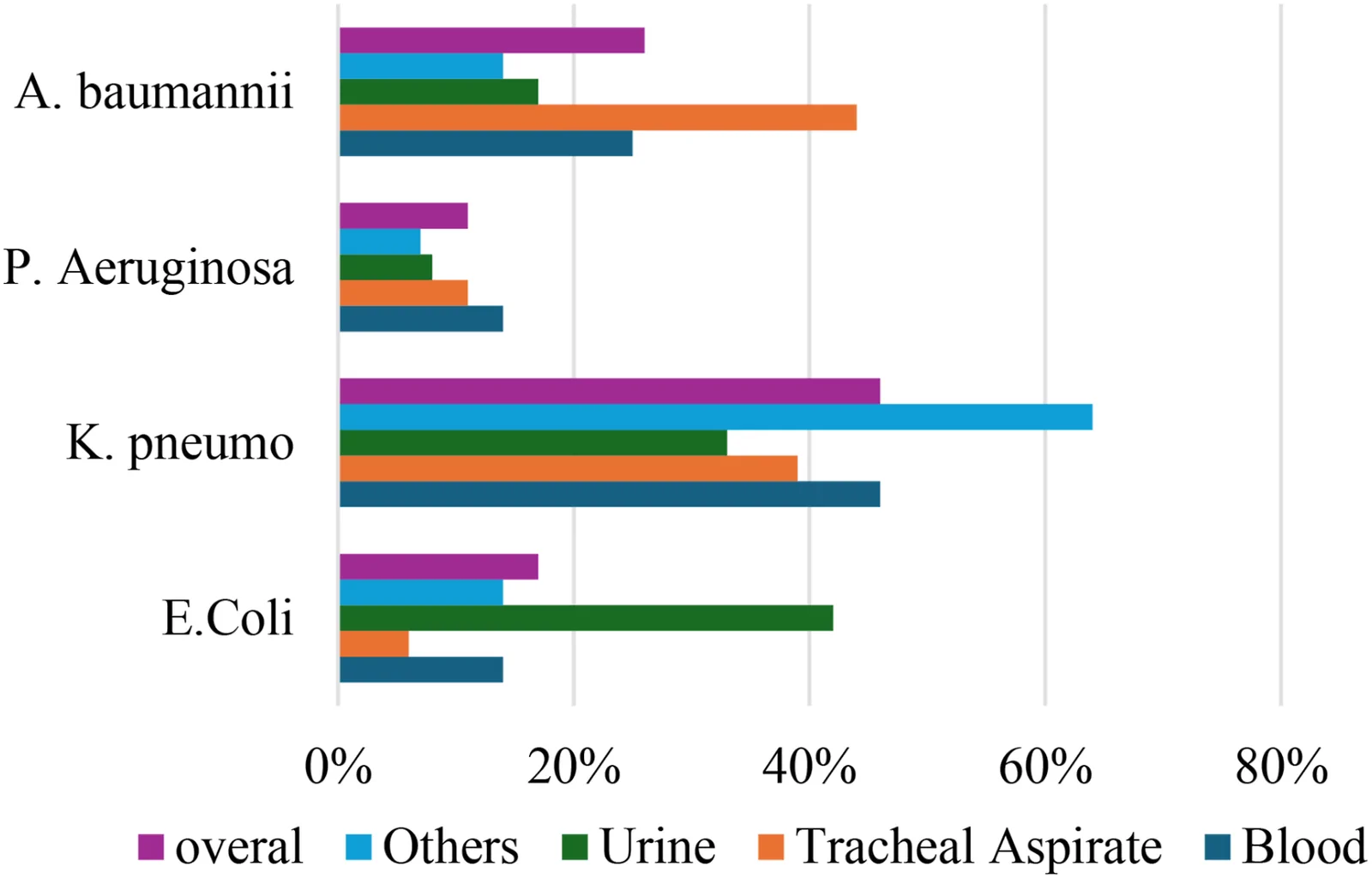
Specimen distribution for resistant pathogens.

Resistance patterns fluctuated over time, with *K. pneumoniae* contributing the largest proportion of resistant isolates in most quarters. A notable peak occurred in quarter 1 of 2024, characterized by increased detection of *A. baumannii* and *P. aeruginosa*. These fluctuations did not appear to correspond with quarterly ICU admission volumes (Figure 4). The proportion of carbapenem-resistant isolates ranged from 11% in quarter 4 of 2023 to 33% in quarter 4 of 2024 (Figure 5).

**Figure 4:**
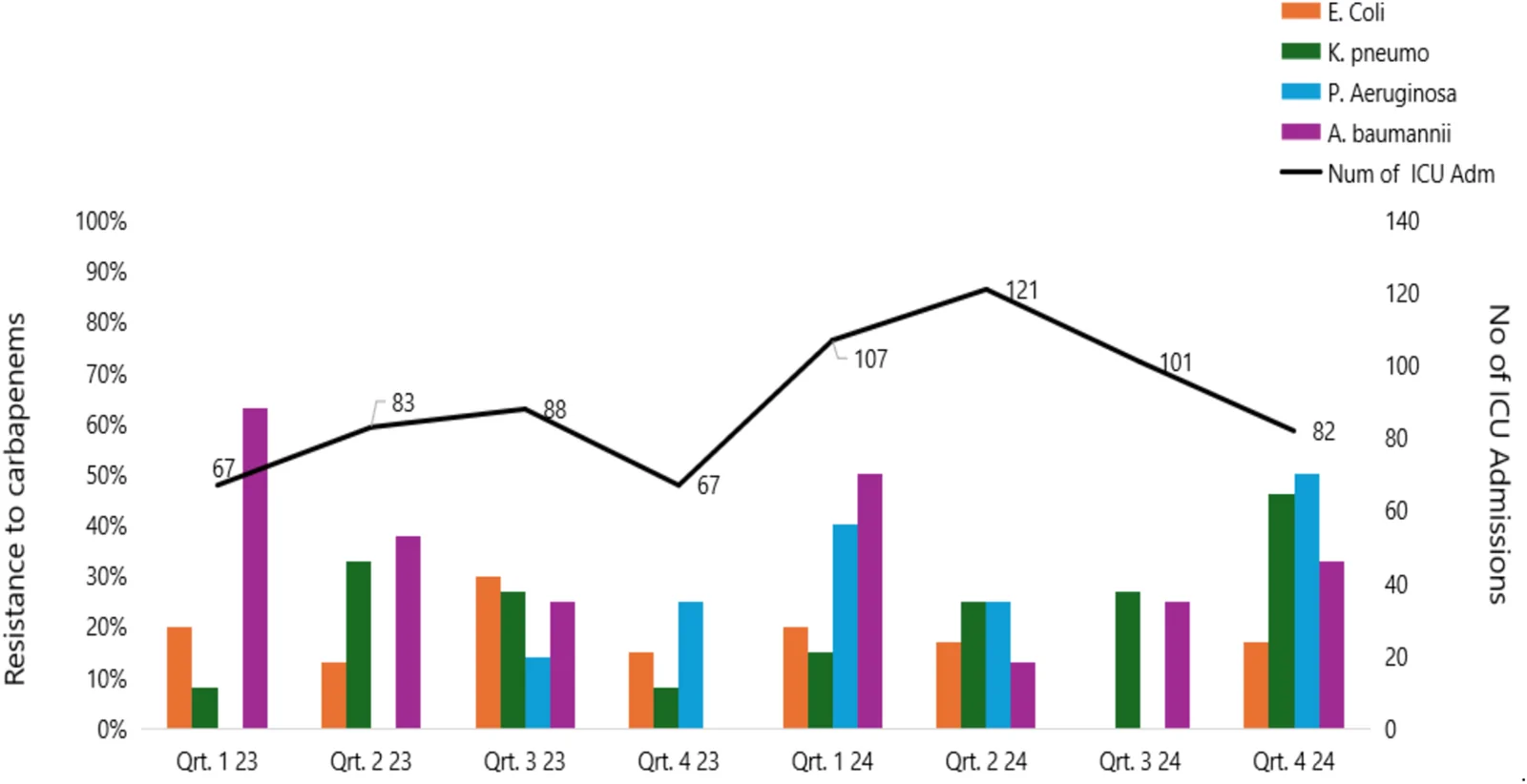
Distribution and trends of carbapenem-resistant organisms. ICU, intensive care unit.

**Figure 5:**
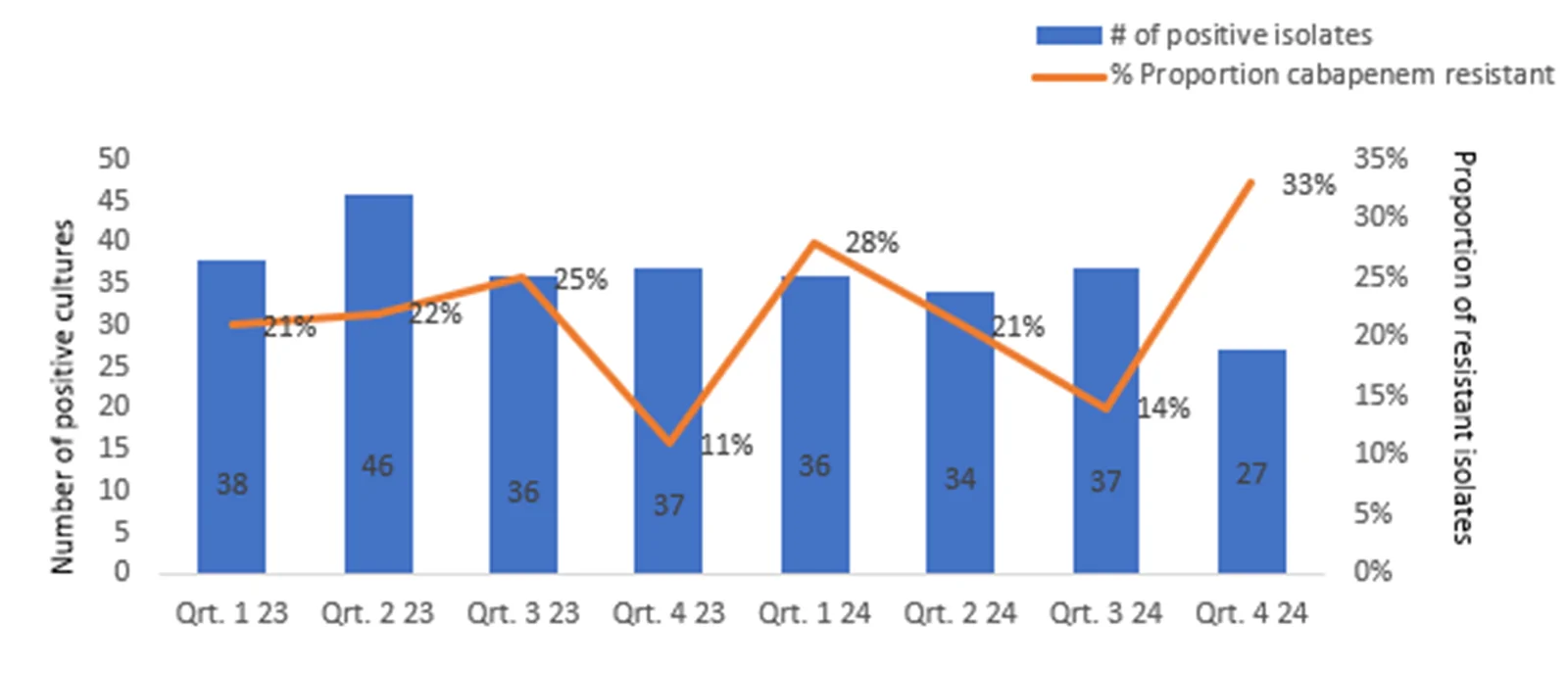
Overall proportion of cabapenem resistance.

Eight spatiotemporal GN-CRO clusters were identified during the study period (Figure 6). Individual ICU bed locations were anonymized as ICU1–ICU11. *A. baumannii* complex demonstrated two clusters and *E. coli* one localized cluster, while *K. pneumoniae* accounted for the majority of identified clusters, including one prolonged cluster. The clusters involved two to five cases and occurred within the predefined 14-day temporal window.

**Figure 6:**
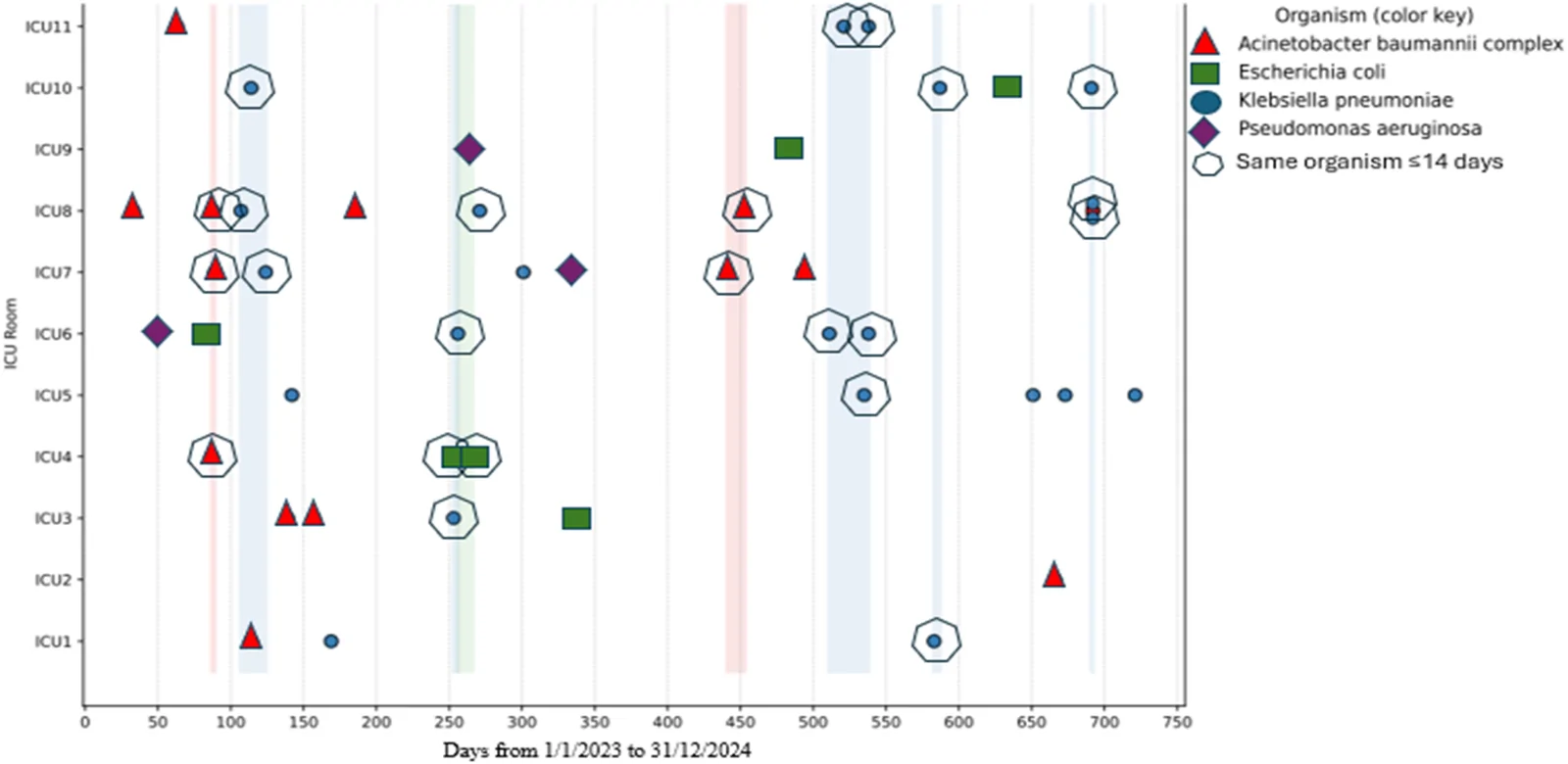
Spatial–temporal scatter plot with 14-day temporal cluster. ICU, intensive care unit.

Eight potential epidemiological cluster signals were identified, with observed counts ranging from two to five isolates compared with the prespecified reference rate of one isolate per month (Table I). Observed-to-expected ratios ranged from 2 to 5. The five-isolate *K. pneumoniae* cluster represented the strongest statistical signal (*P* = 0.003) and was classified as a sustained shift by statistical process control (SPC) analysis. Several three-isolate clusters were also identified as spatial–causal signals based on complementary SPC and spatiotemporal analyses. All clusters classified as spatial–causal or sustained-shift signals showed spatial overlap across ICU bed locations.

**Table I:** Spatial–temporal clusters with Poisson-based and statistical process control–based signal interpretation

Organism	Cluster period (days)	Duration (days)	No. of isolates	Expected count (λ = 1/month)	Observed/Expected ratio	Poisson probability (≥observed)	SPC interpretation	Spatiotemporal overlap (ICU beds)
*Acinetobacterbaumannii* complex	87–89	3	3	≈1	3	≈0.06	Special-cause signal	ICU4, ICU7, ICU8
	441–453	13	2	≈1	2	≈0.18	Alert-level signal	ICU7, ICU8
*Escherichiacoli*	256–266	11	2	≈1	2	≈0.18	Alert-level signal	ICU4
*Klebsiellapneumoniae*	107–124	18	3	≈1	3	≈0.06	Special-cause signal	ICU7, ICU8, ICU10
	253–256	4	2	≈1	2	≈0.18	Alert-level signal	ICU3, ICU6
	511–538	28	5	≈1	5	≈0.003	Sustained shift	ICU5, ICU6, ICU11
	583–587	5	2	≈1	2	≈0.18	Alert-level signal	ICU1, ICU10
	691–692	2	3	≈1	3	≈0.06	Special-cause signal	ICU8, ICU10

SPC, statistical process control; ICU, intensive care unit.

## Discussions

This study demonstrates that automated EHR-based surveillance can lessen the burden of manual data collection and support surveillance activities by detecting potential transmission signals of GN-CROs which can inform subsequent IPC investigations and interventions [16]. These findings are consistent with those of Li *et al.* and Schaumburg, T. *et al.* that demonstrated how timely and accurate electronic surveillance data may reduce the burden of manual surveillance and facilitate more efficient implementation of IPC interventions [12], [17]. *K. pneumoniae* and *A. baumannii* emerged as the predominant carbapenem-resistant pathogens, accounting for the most resistant isolates and the most stable clustering signals during the surveillance period, consistent with regional and global data reporting the same organisms as dominant drivers of ICU-associated carbapenem resistance, particularly in low-resourced settings [5], [18], [19].

The sustained predominance of *K. pneumoniae*, coupled with prolonged and recurrent clustering, suggested potential transmission signals rather than sporadic introductions. This pattern is consistent with special-cause variation, a non-random variation arising from likely identifiable change in the underlying process, such as breakdown in infection prevention practices or increased transmission opportunities, rather than expected background fluctuation. This indicates a shift in the ICU possible transmission process requiring investigation and intervention, rather than routine monitoring alone. Similar SPC-based interpretations have been discussed for use in high-income settings to trigger outbreak investigations, supporting the applicability of these methods in low- and middle-income country contexts [20].

The GN-CRO proportion did not appear to be influenced by fluctuations in ICU admission volumes during the surveillance period. This suggests that increased device use and greater care complexity associated with higher admissions did not translate into amplified transmission within the unit. These findings differ from those reported by Wilson *et al.*, who observed that higher ICU occupancy was associated with increased transmission of resistant organisms. However, admission volume may function as a pressure multiplier in settings where infection prevention resources, staffing ratios, and environmental cleaning capacity are constrained [21].

Months with three or more isolates of the same organism were statistically uncommon, and their convergence with spatial overlap across ICU beds supported their epidemiological plausibility. Recurrent clusters involving adjacent or frequently reused beds may signal localized transmission through shared equipment, environmental reservoirs, or lapses in infection prevention practices. However, these clusters should be interpreted as signals warranting further IPC investigation rather than evidence that EHR-based surveillance reduced transmission or improved patient outcomes. This is consistent with prior findings of a study by Gu, G. Y. *et al.* Demonstrating that prior bed occupants with MDRO infection/colonization could increase the risk of MDRO acquisition in subsequent occupants in the ICU [22].

The observed recurrent seasonal peaks across multiple Gram-negative organisms, including *E. coli* and *A. baumannii* complex, during similar calendar months in consecutive years are consistent with prior work by Stachel *et al.*, who demonstrated that high-risk clusters frequently involved environmentally associated organisms and were best detected through unit-level and healthcare-associated analyses rather than molecular confirmation alone. In their study, suspected transmission events were characterized by shared unit exposure and overlapping healthcare worker contact, even when molecular typing failed to demonstrate clonal relatedness. Similarly, in this study, temporally structured increases occurring across different organisms and overlapping ICU bed locations suggest that potential transmission dynamics may be driven by shared care environments, workflow pressures, or environmental reservoirs rather than single-strain outbreaks. The recurrence of these patterns across seasons further supports the presence of systemic drivers such as staffing strain, patient volume, and acuity, or environmental factors, rather than stochastic variation [6], [23].

From an infection prevention and control perspective, these findings underscore the practical use of automated surveillance in triggering timely, targeted investigations. All identified clusters occurred within CDC-defined temporal thresholds for MDRO investigation, demonstrating alignment between algorithm-based detection and established public health guidance. The ability to link microbiological data with patient location and admission timelines signals rapid detection of potential transmission networks, even without genomic confirmation. This is consistent with prior evidence demonstrating that comprehensive EHR data can extend infection surveillance capabilities in settings with limited infection prevention resources, while NHSN hospital-onset definitions alone identify only 44.4% of healthcare-associated transmission events when compared with whole-genome sequencing surveillance [24], [25].

Another important implication of these findings is that, although molecular typing is valuable for establishing genetic relatedness and supporting the investigation of transmission pathways, observed clustering may arise through multiple mechanisms and should not necessarily be interpreted as evidence of clonal transmission. Epidemiologic surveillance can therefore serve as an important first-line approach for identifying temporal and spatial signals of potential transmission and triggering further investigation and corrective action, particularly in settings where molecular surveillance is limited. Molecular data should consequently be viewed as complementary to, rather than a prerequisite for, epidemiologic detection and infection prevention response [26].

Our study had several limitations. First, while this study highlights the potential of integrating routinely collected EHR and microbiological data for cluster detection, the necessary pre-integration of automated microbiology and EHR-derived clinical data may have introduced harmonization constraints, and implementation feasibility, resource requirements, and scalability across other ICUs or healthcare settings were not formally evaluated. Second, because molecular typing was not performed, the genetic relatedness of the clustered isolates could not be definitively established. Therefore, the observed temporal and spatial clustering should not be interpreted as confirmation of clonal transmission but rather as an epidemiological signal warranting further investigation. Third, because this was a retrospective study, real-time operational use of the surveillance approach was not assessed, prospective implementation would be required to determine whether such signals can generate timely alerts and lead to effective IPC responses. The potential for alert fatigue, particularly if low-specificity or repetitive alerts are generated, was also not evaluated and should be considered in future implementation studies. Additionally, surveillance was based on clinically indicated cultures, which may introduce selection bias as systematic screening for colonization was not performed and asymptomatic carriage may have been missed. As a result, detected clusters likely represent the more severe or clinically apparent end of the transmission spectrum. Also, the analysis was limited to ICU admissions and may not capture transmission events occurring prior to ICU transfer or after discharge. Finally, this study did not include a formal economic evaluation; therefore, the costs associated with developing, maintaining, and implementing EHR-based surveillance, or the potential resource savings compared with conventional surveillance, could not be determined. Also, the spatial analysis was primarily based on ICU bed location and therefore provides information on spatial proximity rather than a complete assessment of transmission pathways. Despite these limitations, the consistency of temporal, spatial, and statistical signals supports the robustness of the findings.

In conclusion, the findings support the potential use of routinely collected EHR and microbiological data for automated detection of epidemiological signals. Integrated electronic surveillance can not only detect meaningful clusters of carbapenem-resistant organisms in the ICU but may also facilitate risk identification that can be useful in preventing infections. An integrated EHR-based approach combining organism tracking, spatiotemporal analysis, and SPC can support the identification of unusual resistance patterns and potential transmission clusters, generating epidemiological signals for further investigation and targeted IPC response. This validates electronic surveillance as a critical tool for infection transmission signals in resource-limited settings, proving that actionable insights can be derived from epidemiological data even without advanced molecular diagnostics. Future studies should assess implementation costs, real-time operational use, impacts on IPC practices, the risk of alert fatigue, personnel requirements, and resource use to determine the cost-effectiveness, feasibility, and scalability of EHR-based surveillance systems in resource-limited settings.

## CRediT authorship contribution statement

**David Odada:** Writing – original draft, Software, Resources, Project administration, Methodology, Investigation, Formal analysis, Data curation, Conceptualization. **Reena Shah:** Writing – review & editing, Writing – original draft, Validation, Supervision, Project administration, Methodology, Funding acquisition, Conceptualization. **Farah Ebrahim:** Writing – review & editing, Writing – original draft, Visualization, Software, Methodology. **Hellen Munyi:** Writing – review & editing, Validation, Supervision. **Annastacia Mbithi:** Writing – review & editing, Resources, Methodology. **Jasmit Shah:** Validation, Supervision, Software, Methodology, Data curation. **Revathi Gunturu:** Validation, Supervision, Project administration, Conceptualization.

## Ethical approval

This study received exemption from informed consent by the Aga Khan University Hospital, Nairobi, Institutional Scientific and Ethics Review Committee, Ref: 2025/ISERC-71 (v2), and was approved to be conducted by the National Commission for Science, Technology and Innovation (NACOSTI), Licence No. NACOSTI/P/25/4176423. All data were de-identified before analysis, with unique identifiers replaced by coded values.

## Funding sources

None declared.

## Conflict of interest statement

None declared.

## References

[1] Kedišaletše M., Phumuzile D., Angela D., Andrew W., Mae N.-F. (2023). Epidemiology, risk factors, and clinical outcomes of carbapenem-resistant enterobacterales in Africa: a systematic review. J Glob Antimicrob Resist.

[2] Loaiza W.M., Ruiz A.K.R., Patiño C.C.O., Vivas M.C. (2023). Bacterial resistance in hospital-acquired infections acquired in the intensive care unit: a systematic review. Acta Med.

[3] Marsh J.W., Mustapha M.M., Griffith M.P., Evans D.R., Ezeonwuka C., Pasculle A.W. (2019). Evolution of outbreak-causing carbapenem-resistant Klebsiella pneumoniae ST258 at a tertiary care hospital over 8 years. mBio.

[4] Mishra A., Dwivedi R., Faure K., Morgan D.J., Cohn J. (2025). Estimated undertreatment of carbapenem-resistant Gram-negative bacterial infections in eight low-income and middle-income countries: a modelling study. Lancet Infect Dis.

[5] Maina J.W., Mutua J.M., Musyoki A.M. (2024). Carbapenem-resistant gram-negative bacterial infections and risk factors for acquisition in a Kenyan intensive care unit. BMC Infect Dis.

[6] Saharman Y.R., Karuniawati A., Severin J.A., Verbrugh H.A. (2021). Infections and antimicrobial resistance in intensive care units in lower-middle income countries: a scoping review. Antimicrob Resist Infect Control.

[7] Rosenthal V.D., Yin R., Lu Y., Rodrigues C., Myatra S.N., Kharbanda M. (2023). The impact of healthcare-associated infections on mortality in ICU: A prospective study in Asia, Africa, Eastern Europe, Latin America, and the Middle East. Am J Infect Control.

[8] Bunduki G.K., Masoamphambe E., Fox T., Musaya J., Musicha P., Feasey N. (2024). Prevalence, risk factors, and antimicrobial resistance of endemic healthcare-associated infections in Africa: a systematic review and meta-analysis. BMC Infect Dis.

[9] Nishida S., Ono Y. (2025). Genomic plasticity of extensively drug-resistant and multidrug-resistant Acinetobacter baumannii ST208 isolates from a fatal outbreak. J Infect Public Health.

[10] Stachel A., Pinto G., Stelling J., Fulmer Y., Shopsin B., Inglima K. (2017). Implementation and evaluation of an automated surveillance system to detect hospital outbreak. Am J Infect Control.

[11] Miller J.K., Chen J., Sundermann A., Marsh J.W., Saul M.I., Shutt K.A. (2019). Statistical outbreak detection by joining medical records and pathogen similarity. J Biomed Inf.

[12] Schaumburg T., Köhler N., Breitenstein Y., Kolbe-Busch S., Hasenclever D., Chaberny I.F. (2023). ICU infection surveillance can be based on electronic routine data: results of a case study. BMC Infect Dis.

[13] Sundermann A.J., Miller J.K., Marsh J.W., Saul M.I., Shutt K.A., Pacey M. (2019). Automated data mining of the electronic health record for investigation of healthcare-associated outbreaks. Infect Control Hosp Epidemiol.

[14] Streefkerk H.R.A., Verkooijen R.P., Bramer W.M., Verbrugh H.A. (2020). Electronically assisted surveillance systems of healthcare-associated infections: a systematic review. Euro Surveill.

[15] Sips M.E., Bonten M.J.M., van Mourik M.S.M. (2017). Automated surveillance of healthcare-associated infections: state of the art. Curr Opin Infect Dis.

[16] Reilly J.S., McCoubrey J., Cole S., Khan A., Cook B. (2015). Integrating intensive care unit (ICU) surveillance into an ICU clinical care electronic system. J Hosp Infect.

[17] Li X., Cai P., Zhong H., Yan C., Wen R. (2024). Uses of a real-time automatic nosocomial infection surveillance system to support prevention and control of hospital-acquired infections in the ICU. Front Public Health.

[18] Kerimoglu E., Catak T., Kilinc A. (2025). Evaluation of Infections Caused by Carbapenem-Resistant Pseudomonas aeruginosa, Acinetobacter baumannii, and Klebsiella pneumoniae in an Intensive Care Unit: A Retrospective Study. Antibiotics.

[19] Xie Y., Tan X., Wang W., Hou B., Mao M., Niu X. (2025). Bacteriological Spectrum and Drug Resistance Among Patients Associated With Bloodstream Infection in Intensive Care Units in the Affiliated Hospital of Jiaxing University From 2021 to 2023. Can J Infect Dis Med Microbiol.

[20] Qiu P. (2024).

[21] Wilson T., Nolte D., Omar S. (2024). Bed occupancy and nosocomial infections in the intensive care unit: A retrospective observational study in a tertiary hospital. Southern Afr J Crit Care.

[22] Gu G.Y., Chen M., Pan J.C., Xiong X.L. (2023). Risk of multi-drug-resistant organism acquisition from prior bed occupants in the intensive care unit: a meta-analysis. J Hosp Infect.

[23] Stachel A., Pinto G., Stelling J., Fulmer Y., Shopsin B., Inglima K. (2017). Implementation and evaluation of an automated surveillance system to detect hospital outbreak. Am J Infect Control.

[24] Sundermann A.J., Penzelik J., Ayres A., Snyder G.M., Harrison L.H. (2023). Sensitivity of National Healthcare Safety Network definitions to capture healthcare-associated transmission identified by whole-genome sequencing surveillance. Infect Control Hosp Epidemiol.

[25] Branch-Elliman W., Sundermann A.J., Wiens J., Shenoy E.S. (2023). Leveraging electronic data to expand infection detection beyond traditional settings and definitions (Part II/III). Antimicrob Steward Healthc Epidemiol.

[26] Leclère B., Buckeridge D.L., Boëlle P.Y., Astagneau P., Lepelletier D. (2017). Automated detection of hospital outbreaks: A systematic review of methods. PLoS One.

